# Avoidable Portion of Tobacco-Attributable Acute Care Hospital Days and Its Cost Due to Implementation of Different Intervention Strategies in Canada

**DOI:** 10.3390/ijerph6082179

**Published:** 2009-08-06

**Authors:** Svetlana Popova, Jayadeep Patra, Jürgen Rehm

**Affiliations:** 1 Public Health and Regulatory Policies, Centre for Addiction and Mental Health, Toronto, Canada; E-Mails: jaydeep_patra@gmail.com (J.P.); jtrehm@aol.com (J.R.); 2 Dalla Lana School of Public Health, University of Toronto, Canada; 3 Factor-Inwentash Faculty of Social Work, University of Toronto, Canada; 4 Epidemiological Research Unit, Klinische Psychologie & Psychotherapie, Technische Universität Dresden, Germany

**Keywords:** tobacco smoking, public health, health effects, lung cancer, cardiovascular disease, smoking cessation, tobacco taxation, tobacco consumption reduction

## Abstract

The impact of four effective population-based interventions, focusing on individual behavioural change and aimed at reducing tobacco-attributable morbidity, was assessed by modeling with respect to effects on reducing prevalence rates of cigarette smoking, population-attributable fractions, reductions of disease-specific morbidity and its cost for Canada. Results revealed that an implementation of a combination of four tobacco policy interventions would result in a savings of 33,307 acute care hospital days, which translates to a cost savings of about $37 million per year in Canada. Assuming 40% coverage rate for all individually based interventions, the two most effective interventions, in terms of avoidable burden due to morbidity, would be nicotine replacement therapy and physicians’ advice, followed by individual behavioural counselling and increasing taxes by 10%. Although a sizable reduction in the number of hospital days and accumulated costs could be achieved, overall these interventions would reduce less than 3% of all tobacco-attributable costs in Canada.

## 1. Introduction

Smoking is one of the most important risk factors for the burden of disease. Tobacco use is responsible for high levels of morbidity and mortality. Smoking causes a substantially increased risk of mortality from lung cancer, upper aerodigestive cancer, several other cancers, heart disease, stroke, chronic respiratory disease and a range of other medical causes [1]. In the developed world in the year 2000, smoking was reported to be the risk factor with the largest attributable mortality and attributable disability adjusted life years (DALYS); specifically, 12.2% of all DALYS were attributed to this risk factor [2].

The second Canadian Cost Study indicated that the social costs for substance abuse in Canada are high, with a cost of $39.8 billion in 2002 [3,4]. The economic costs of tobacco abuse were the highest among all substances, with a cost of $17.0 billion, which represented 42.7% of the total substance abuse costs in Canada.

Given the evident overload of tobacco-attributable social burden, tobacco control measures have gained more importance. The use of cost-effective tobacco control measures is the key to further reduce the burden of tobacco smoking [5,6]; for the field of substance abuse see [7–9]. Analysis of avoidable burden and avoidable costs of tobacco-attributable morbidity in Canada was thus necessary in finding such effective measures.

## 2. Methodology

### 2.1. Selection of Interventions

The intervention selection was undertaken in two steps:

1) A collection of evidence for the most common interventions via a search of meta-analyses with a special emphasis on Cochrane Reviews; and 2) an expert consultation to select the best fitting types of interventions for Canada.

### 2.2. Methodological Considerations for Statistical Modeling

Based on previous publications [10–12], we decided to model the impact of different interventions in terms of burden of disease. This procedure can be justified by the fact that for tobacco abuse (The term “abuse” here is used in the economical definition and does not necessarily effect the psychiatric definition of DSM-IV)–contrary to alcohol abuse and illicit drugs–the overwhelming majority of direct costs materializes in health care [3,13,14].

The usual epidemiological model, as defined by burden of disease studies, especially on the international level [2,15–17], operates with one-dimensional risk factors and foresees the following steps:

→ Estimation of population disease with sex and age specific population-attributable fractions, in the case of tobacco with smoking-attributable fractions (SAF).
→ Based on SAF, tobacco-attributable morbidity expressed in the number of acute care hospital days.

### 2.3. Computing Smoking-Attributable Fractions

The contribution of a risk factor to disease or mortality, relative to some alternative exposure scenario (i.e., PAF, defined as the proportional reduction in population disease or mortality that would occur if exposure to the risk factor were reduced to an alternative exposure scenario, *ceteris paribus* [18,19]), is given by the generalized “potential impact fraction” in Equation 1, or its discrete version when the exposure variable is categorical [19–21]:

(1) $$PIF=\frac{\int_{x=0}^{m}RR(x)P(x)\;dx-\int_{x=0}^{m}RR(x)P^{\prime }(x)\;dx}{\int_{x=0}^{m}RR(x)P(x)\;dx}$$

*RR*(*x*): relative risk at exposure level *x*
*P*(*x*): population distribution of exposure
*P*′(*x*): counterfactual distribution of exposure (often 0 = no exposure for tobacco)
*m*: maximum exposure level

Since most diseases are multifactorial (caused by a multiple number of risk factors), and because some risk factors act through other more proximal factors, population-attributable fractions for multiple risk factors for the same disease can add up to more than 100% [22,23]. For example, some of the cardiovascular disease events may be due to a combination of smoking, physical inactivity and an inadequate intake of fruits and vegetables (all acting partially through obesity, cholesterol, and blood pressure). Such cases would be attributed to all of these risk factors. While the lack of additivity may seem problematic initially, multiple-causality offers the opportunity to tailor prevention based on the availability and the cost of the interventions. In terms of tobacco interventions, this means that the projected morbidity gains will be achieved through constellations, in which some of the gains could also be achieved by other interventions; e.g. the morbidity reduction of tobacco taxation on CHD could in part be achieved by improving physical fitness in the population. To estimate tobacco-attributable morbidity, SAFs were calculated using the discrete version of Equation 1.

### 2.4. Smoking Risk Relations

As indicated by Equation 1, the calculation of tobacco-attributable morbidity was based on the combination of relative risks (RRs) and prevalence of exposure. The selection of tobacco-related diseases and causes of morbidity relied on the comprehensive reviews by the International Agency for Research on Cancer [24] and the U.S. Government [1]. These reviews consider the following criteria in judgments of causality: consistency, strength of association, specificity, temporality, coherence, dose-response, and experimental evidence. Once identified, the conditions were translated into corresponding International Classification of Diseases (ICD) 9 and 10 codes. The list of these conditions is reported in Table 1.

The RRs were abstracted from a comprehensive review of the determinants of health prepared by the Australian Government, which contained systematic meta-analyses of the health effects of tobacco smoking [25]. The RRs for ex-smokers and current smokers are listed in Table 1. The SAFs for morbidity were calculated by combining the RRs with the exposure prevalence.

### 2.5. Prevalence of Smoking in Canada

Smoking prevalence for different levels of smoking consumption for Canada, as a whole, were obtained from the Canadian Community Health Survey 2003 (CCHS cycle 2.1), a population based representative survey conducted by Statistics Canada [26]. All prevalence estimates were sex- and age group-specific. However, the categorization of smoking status varied by specified disease and were based on the RRs available in the meta-analyses. For each disease, for which the identified meta-analysis included dose-response-specific RR, prevalence estimates were also dose-specific (e.g., never, former, current, 1–14, 15–24, 25+ cigarettes per day). Current smokers, those who reported occasional smoking or daily smoking, were further categorized by the number of cigarettes smoked per day, when sufficient information existed to do so.

In order to model smoking behaviour in Canada with pressure towards reducing smoking rates, we assumed a scenario based on the literature; e.g. trends observed in regions of North America and Australia featuring intense efforts to reduce tobacco related harm. These scenarios were based on the following (see also [27]):

- Yearly quitting rates of 10%;
- The assumption that 80% of smokers wanted to quit;
- The assumption of an annual incidence rate (new cases of smokers before and after intervention in the specified year) of 0.46% for current female non-smokers, and prevalence proportionate incidence rate for males.

### 2.6. Morbidity Data

The number of acute care hospital days in Canada for 2002 was obtained from the Canadian Institute for Health Information (CIHI), on the national and the provincial level according to ICD-10 codes. The national level data was composed of only seven provinces and two territories (Alberta, British Columbia, Newfoundland, Northwest Territories, Nova Scotia, Ontario, Prince Edward Island, Saskatchewan, and Yukon Territory). For the national level, data was provided for each disease condition, as well as for each sex and age 20+. Based on these figures, the data for Canada, as a whole, were estimated using the total population: the disease-specific rate of occurrence observed in the data provided was applied to the total population of Canada to obtain the estimated number of disease-specific occurrences.

The Hospital Morbidity Database (HMDB), held by CIHI, captures information on patients separated through discharge or death from acute care facilities in Canada. This database provides national data on acute care hospitalizations by diagnoses and procedures excluding day procedures (e.g., day surgeries), outpatient, and emergency department visits. The HMDB also includes data on newborns but not stillborns and cadaveric donor “discharges”. Also to note, figures are based on facility geography, that is, where the hospital is located, thus possible non-Canadians may be included. Additionally, the statistics reflect the number of hospitalizations, which is somewhat higher than the number of individuals diagnosed since individuals with multiple admissions during a single year would be counted more than once.

The number of hospital days (i.e., length of stay) is associated with the condition coded as Most Responsible Diagnosis (MRD) on the patient’s hospital record. This means that the MRD accounts for the most of the days a patient stays in a hospital. A diagnosis of MRD is described as the most significant condition of the patients’ that is responsible for his/her stay in the hospital. When multiple diagnoses are classified as the most responsible, coders are instructed to code the diagnosis responsible for the longest length of stay [28]. As the hospital days based on the MRD may overlap in cases with more than one MRD, the calculated hospital days had to be adjusted to the overall hospital days in Canada. This adjustment implied a province-specific application of a shrinkage factor, derived by dividing the number of hospital days in a province by the number of MRD hospital days in the same province.

### 2.7. Estimating Avoidable Morbidity and Its Cost

The baseline scenario costs of acute care hospitalizations were estimated by multiplying the SAF for conditions known to be affected or caused by tobacco smoking by the aggregate number of acute care hospital days for each condition by age and sex. These figures were then multiplied by the per diem cost of acute care hospital days, by condition and by province or territory, using costs obtained from a variety of sources (Table 2, [3,29]). The total national figures were then calculated by aggregating the total costs due to tobacco-attributable conditions across provinces and territories.

To compute the avoidable costs of the interventions related to acute care hospital days, we applied the estimated percentage changes in the SAF caused by the intervention for each tobacco-attributable condition to the baseline figures. We, thereby, obtained the changes in costs for all tobacco-attributable conditions, which were then aggregated to provide the estimated avoidable cost of each intervention.

## 3. Results

### 3.1. Collection of Evidence for Most Common Interventions

Fifty-one systematic reviews were found with respect to the effectiveness of specific smoking-related interventions. Seven experts were contacted to identify four evidence-based intervention strategies to reduce tobacco-attributable morbidity in Canada. As a result, the following interventions were selected based on the feedback of the experts:

#### a) Public policy interventions:

##### 1) Price increase

There is a strong link between the price of cigarettes and its consumption: increases in the cost of cigarettes to the consumer will decrease consumption rates and, therefore, decrease tobacco-related problem rates.

#### b) Interventions focusing on individual behavioural change (counselling, brief advice, therapy):

##### 2) Individual behavioural counselling (IBC) for smoking cessation

IBC was defined as a face-to-face encounter between a smoking patient and a counsellor trained in assisting smoking cessation. This excludes counselling delivered by doctors and nurses as part of clinical care.

##### 3) Nicotine replacement therapy (NRT) for smoking cessation

NRT included chewing gum, transdermal patches, nasal spray, inhalers (a cigarette-like device which delivers nicotine to the buccal mucosa by sucking) and tablets or lozenges.

##### 4) Physician advice for smoking cessation

Physician advice to stop smoking was defined as verbal instructions from the physician with a ‘stop smoking’ message irrespective of whether or not information was provided about the harmful effects of smoking. Advise as part of multifactorial lifestyle counselling (e.g., including dietary and exercise advice) was excluded. Therapists were physicians, or physicians supported by another healthcare worker.

Table 3 provides a summary of the effectiveness of the selected interventions.

### 3.2. Exposure

**Figure 1.**
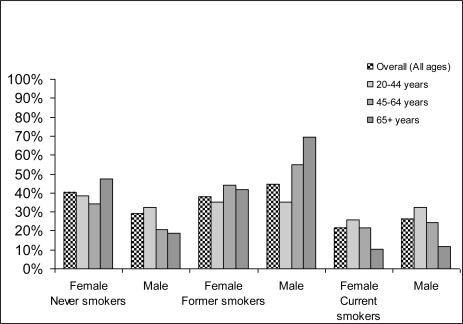
Prevalence in percentage of different smoking categories by gender and age in Canada in 2002. Source: Canadian Community Health Survey (CCHS) 2003 [26]

Figure 1 provides an overview of exposure to smoking in Canada by sex and age. As expected, men smoked more than women on average, and smoking prevalence decreased with age.

### 3.3. Tobacco-Attributable Morbidity in Canada 2002

Overall, 1,408,252 hospital days were estimated to be attributable to tobacco (815,059 for men and 593,193 for women) in Canadian population over 20 years old in 2002. This constitutes 6.6% of all hospital days in acute care hospitals in Canada (men: 8.5%; women: 5.0%).

The two single disease categories ischaemic heart disease (IHD) and lung cancer made up the majority of tobacco-attributable acute care hospital days - about 40% (35.9% in men, 45.3% in women). Specifically, IHD accounted for 25% of the total tobacco-attributable hospital days (350,793 hospital days; men: 173,418; women: 177,375). The next largest single category was lung cancer (15%; 209,627 hospital days, men: 118,788, women: 90,839).

### 3.4. Effectiveness of Interventions

Table 4 translates the effects of selected interventions into the common metric of smoking prevalence rates in the Canadian adult population (operationalized as all inhabitants 15 years and older).

### 3.5. Avoidable Morbidity and Its Cost in Canada

Table 5 shows the effectiveness of interventions on morbidity, measured as acute care hospital days, in Canada. The results revealed that an implementation of the four aforementioned interventions related to tobacco policy combined would result in a savings of 33,307 acute care hospital days.

This would result in cost savings of about $37 million in Canada per year (Table 6).

**Figure 2.**
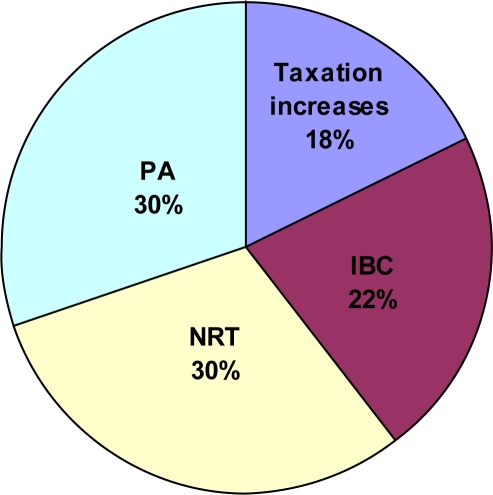
Interventions and their impact on tobacco-attributable acute hospital days (all cause), 20+ years in Canada (2002). IBC–Individual behavioural counselling NRT–Nicotine replacement therapy PA–Physician’s advice

The most effective intervention in terms of avoidable burden due to morbidity was nicotine replacement therapy and physician’s advice (savings more than $11 million per each intervention, 60% of total savings), followed by individual behavioural counselling (more than $8 million per year, 22% of total savings) and increasing taxes (more than $6.5 million per year, 18% of total savings; Tables 5, 6 and Figure 2).

Additionally, this study estimated the effect of the interventions on the burden and the cost of two of the biggest contributors: IHD and lung cancer. The results revealed that an implementation of the four interventions related to tobacco policy combined would result in a savings of 20,264 acute care hospital days due to IHD and 1,052 acute care hospital days due to lung cancer in Canada. This would result in cost savings of about $22.5 million (57% of total savings) for IHD and more than $1.2 million for lung cancer in Canada per year.

### 3.6. Limitations and Conclusion of the Study

This study has several limitations. First, the effects of all interventions were modeled as if they occurred instantaneously, therefore the combined effect of four interventions is possibly overestimated. In addition, the study did not estimate over what periods of time that these benefits in morbidity and cost would be achievable. Furthermore, the choice of implementing a single intervention or combined interventions serves as lower and upper estimates, respectively, for this study.

The study also overestimates the effects on chronic health conditions that are solely attributable to tobacco. For example, if some intervention could reduce tobacco consumption to zero at a certain point in time, tobacco-related disease burden would not be zero immediately thereafter. Instead, some burden of disease would persist due to previous tobacco consumption. For instance, there will be some people already having tobacco-attributable lung cancer and some people may even develop new lung cancer or other cancer in the future based on their past tobacco exposure.

The study also did not take into consideration the effects of the ongoing interventions aimed to prevent multifactorial diseases such as cardiovascular diseases and cancer.

In addition, current estimates of avoidable acute care hospital days due to tobacco use and its cost do not reflect the rates of return that the society might achieve. In order to compute the potential rates of return on expenditure, it is necessarily to conduct a cost benefit analysis.

In this study only four exemplary interventions were modeled as a demonstration of the possibility of improving population health and saving public health expenditures associated with tobacco smoking. There are many more effective population-based intervention and interventions focusing on individual behavioural change which would further reduce tobacco-attributable burden and its associated costs. It is our hopes that this study can positively influence the decision making on tobacco control in Canada.

## Figures and Tables

**Table 1.** Tobacco-attributable conditions included in this study and relative risks from English *et al.* [25].

**Cause of morbidity**	**ICD 9**	**ICD10**	**RRs (Source: [25])**		**Comments**
			**FS**	**CS**	
Lip, oral and pharyngeal cancer	140–149	C00–C14	1.76	4.55	RRs for codes: 140–141, 143–149
Esophageal cancer	150	C15	1.79	4.01	
Stomach cancer	151	C16	1.11	1.41	
Liver cancer	155	C22	1.07	1.71	
Pancreatic cancer	157	C25	1.15	1.86	
Laryngeal cancer	161	C32	2.86	7.48	
Lung cancer – m	162	C33–C34	6.75	13.0	
Lung cancer – w	-	-	5.07	11.4	
Cervical cancer	180	C53	1.31	1.75	RRs for codes: 180, 233.1
Bladder cancer	188	C67	1.66	2.72	
Kidney, other urinary cancer	189	C64–C66, C68	1.61	1.64	Renal parenchymal carcinoma −189.0
Leukemia	204–208	C91–C95	1.21	1.01	
Parkinson disease	332	G20–G21	0.57	0.57	
Ischaemic heart disease <65	410–414	I20–I25	1.45	3.06	
Ischaemic heart disease -m 65+	-	-	0.93	1.67	
Ischaemic heart disease -w 65+	-	-	1.22	1.67	
Other heart diseases	390–398, 415–417, 420–429	I00–I09, I26–I51	NA	NA	
Cerebrovascular disease <65	430–438	I60–I69	1.30	3.12	
Cerebrovascular disease 65+	-	-	1.15	1.65	
Atherosclerosis	440	I70	NA	NA	
Other arterial diseases	441–448	I71–I78	NA	NA	
Atherosclerosis and other arterial diseases	440–448	I70–I78	1.82	2.54	
Pneumonia	480–487	J10–J18	1.29	1.47	
Bronchitis, emphysema	490–492	J40–J43	NA	NA	
Chronic airways obstruction	496	J44	NA	NA	
COPD	490–492, 496	J40–J44	6.70	9.80	
Peptic ulcer	531–534	K25–K27	2.24	2.07	
Crohn disease – m	555	K50	1.92	1.92	
Crohn disease – w	-	-	1.60	3.27	
Ulcerative colitis	556	K51	1.71	0.63	
Fire injury	E890–E899	X00–X09	NA	NA	TAF = 23%

M–men; W–women

FS–former smokers; CS–current smokers

**Table 2.** Total cost per night in acute care hospital per province and territory, and Canada, 2002.

**Provinces**	**Average cost per night $**

Alberta	1,311
British Columbia	1,524
Manitoba	1,346
New Brunswick	1,284
Newfoundland	1,455
Nova Scotia	1,217
Ontario	1,045
Prince Edward Island	798
Quebec	990
Saskatchewan	1,263
Northwest Territory	2,177
Nunavut	SUB
Yukon Territory	883
CANADA	1,109

SUB = substitution (average cost of Northwest Territories was substituted)

**Table 3.** Interventions and their effectiveness.

**Type of intervention**	**Effect**	**Reference**
***Public policy interventions***		
Taxation and price increases	2% prevalence reduction in smokers associated with 10% increase in price, based on meta-analysis for high income countries.	[30]
***Interventions focusing on individual behavioural change***		
Individual behavioural counselling for smoking cessation (non-medical) Nicotine replacement therapy for smoking cessation Physician advice for smoking cessation	Cessation ratio of 1.56 (95% CI: 1.32–1.84) compared to controls without intervention Cessation ratio of 1.58 (95% CI: 1.50–1.66) compared to controls without intervention Cessation ratio of 1.66 (95% CI: 1.42–1.94) compared to controls without intervention	[31] [32] [33]

**Table 4.** Detailed results of effectiveness of different interventions for smoking cessation on prevalence of smoking in Canada (2002).

**Overall (all ages >15)**			**15–19 Yrs**		**20–44 Yrs**		**45–64 Yrs**		**65+ Yrs**	
**M%**		**W%**	**M%**	**W%**	**M%**	**W%**	**M%**	**W%**	**M%**	**W%**
**Natural course plus quitters plus new beginners = baseline scenario**										
**NS**	29.1	40.4	58.3	59.2	32.4	38.7	20.8	34.2	18.8	47.6
**FS**	44.6	37.8	20.0	18.6	35.1	35.3	54.7	44.1	69.7	41.9
**CS**	26.3	21.8	21.8	22.2	32.5	26.0	24.5	21.7	11.5	10.5
	100.0	100.0	100.0	100.0	100.0	100.0	100.0	100.0	100.0	100.0
**Taxation change to result in price increases of 10%**										
**FS**	45.1	38.2	20.4	19.0	35.8	35.8	55.2	44.5	69.8	42.2
**CS**	25.8	21.4	21.3	21.7	31.8	25.5	24.0	21.3	11.4	10.2
			100.0	100.0	100.0	100.0	100.0	100.0	100.0	100.0
**Individual behavioural counselling (assuming 40% coverage based on those willing to quit)**										
**FS**	45.2	38.3	20.5	19.0	35.9	35.9	55.3	44.6	69.9	42.2
**CS**	25.7	21.3	21.3	21.7	31.7	25.4	23.9	21.1	11.3	10.2
			100.0	100.0	100.0	100.0	100.0	100.0	100.0	100.0
**NRT (assuming 40% coverage based on those willing to quit)**										
**FS**	45.4	38.5	20.6	19.3	36.2	36.1	55.5	44.9	70.0	42.4
**CS**	25.4	21.1	21.1	21.5	31.4	25.2	23.7	20.9	11.2	10.1
			100.0	100.0	100.0	100.0	100.0	100.0	100.0	100.0
**Physician’s advice (assuming 40% coverage based on those willing to quit)**										
**FS**	45.4	38.5	20.6	19.2	36.1	36.1	55.5	44.9	70.0	42.4
**CS**	25.5	21.1	21.1	21.5	31.5	25.2	23.7	20.9	11.2	10.1
			100.0	100.0	100.0	100.0	100.0	100.0	100.0	100.0

NS–Never smokers

FS–Former smokers

CS–Current smokers

M–men; W–women

**Table 5.** Interventions and their impact on tobacco-attributable acute hospital days (all cause), 20+ years in Canada (2002).

**All cause**	**20–44 Yrs**		**45–64 Yrs**		**65+Yrs**		**20+ Yrs**					
	**M**	**W**	**M**	**W**	**M**	**W**	**M**	**W**	**Total**			
Natural course plus quitters plus new beginners = baseline scenario	27,789	11,155	241,401	88,257	545,869	493,782	815,059	593,193	1,408,252			
**Various Intervention Scenarios**										**Tobacco-attributable acute hospital days saved**		
										**M**	**W**	**Total**
Taxation change to result in price increases of 10%	27,635	11,102	240,316	87,898	543,801	491,614	811,753	590,614	1,402,367	3,306	2,579	5,885
IBC*	27,613	11,091	240,098	87,808	542,765	491,614	810,477	590,513	1,400,990	4,582	2,680	7,262
NRT*	27,546	11,069	239,661	87,627	541,729	490,528	808,936	589,225	1,398,161	6,123	3,968	10,091
PA*	27,569	11,069	239,661	87,627	541,729	490,528	808,958	589,225	1,398,183	6,101	3,968	10,069
ALL: taxation, IBC, NRT, PA	-	-	-	-	-	-	-	-	-	**20,111**	**13,196**	**33,307**

* assuming 40% coverage based on those willing to quit

IBC–Individual behavioural counselling

NRT–Nicotine replacement therapy

PA–Physician’s advice

M–men; W–women

Numbers may not add up because of rounding (all numbers are based on smoking attributable fractions and thus have decimals)

**Table 6.** Net savings of tobacco-attributable cost (CND $) due to implementation of selected interventions in Canada (2002).

**Selected Interventions**	**M**	**W**	**TOTAL**
***Baseline***	*903,900,431*	*657,851,037*	*1,561,751,468*
**Taxation change to result in price increases of 10%**	900,234,077	654,990,926	1,555,225,003
Difference between baseline and this intervention*	3,666,354	2,860,111	6,526,465
Relative change between baseline and this intervention	0.4%	0.4%	0.4%
**IBC (assuming 40% coverage based on those willing to quit)**	898,818,993	654,878,917	1,553,697,910
Difference between baseline and this intervention*	5,081,438	2,972,120	8,053,558
Relative change between baseline and this intervention	0.6%	0.5%	0.5%
**NRT (assuming 40% coverage based on those willing to quit)**	897,110,024	653,450,525	1,550,560,549
Difference between baseline and this intervention*	6,790,407	4,400,512	11,190,919
Relative change between baseline and this intervention	0.8%	0.7%	0.7%
**PA (assuming 40% coverage based on those willing to quit)**	897,134,422	653,450,525	1,550,584,947
Difference between baseline and this intervention*	6,766,009	4,400,512	11,166,521
Relative change between baseline and this intervention	0.7%	0.7%	0.7%
**Difference between baseline and all interventions**	**22,304,208**	**14,633,255**	**36,937,463**
**Relative change between baseline and all interventions**	**2.5%**	**2.2%**	**2.4%**

* Avoidable cost

IBC–Individual behavioural counselling

NRT–Nicotine replacement therapy

PA–Physician’s advice

M–men; W–women
